# Genetic susceptibility to dyslipidemia and incidence of cardiovascular disease depending on a diet quality index in the Malmö Diet and Cancer cohort

**DOI:** 10.1186/s12263-016-0536-0

**Published:** 2016-07-07

**Authors:** Sophie Hellstrand, Ulrika Ericson, Christina-Alexandra Schulz, Isabel Drake, Bo Gullberg, Bo Hedblad, Gunnar Engström, Marju Orho-Melander, Emily Sonestedt

**Affiliations:** 1 Diabetes and Cardiovascular Disease—Genetic Epidemiology, Department of Clinical Sciences Malmö, Lund University, Jan Waldenströms gata 35, SE-20502 Malmö, Sweden; 2 Nutritional Epidemiology, Department of Clinical Sciences Malmö, Lund University, Jan Waldenströms gata 35, SE-20502 Malmö, Sweden; 3 Cardiovascular Epidemiology, Department of Clinical Sciences Malmö, Lund University, Jan Waldenströms gata 35, SE-20502 Malmö, Sweden

**Keywords:** Cholesterol, Epidemiology, Lipoproteins, Nutrition, Triglycerides

## Abstract

**Background:**

By taking diet quality into account, we may clarify the relationship between genetically elevated triglycerides (TG) and low-density lipoprotein-cholesterol (LDL-C), and better understand the inconsistent results regarding genetically elevated high-density lipoprotein-cholesterol (HDL-C), and cardiovascular disease (CVD) risk.

**Methods:**

We included 24,799 participants (62 % women, age 44–74 years) from the Malmö Diet and Cancer cohort. During a mean follow-up time of 15 years, 3068 incident CVD cases (1814 coronary and 1254 ischemic stroke) were identified. Genetic risk scores (GRSs) were constructed by combining 80 validated genetic variants associated with higher TG and LDL-C or lower HDL-C. The participants’ dietary intake, assessed by a modified diet history method, was ranked according to a diet quality index that included six dietary components: saturated fat, polyunsaturated fat, fish, fiber, fruit and vegetables, and sucrose.

**Results:**

The GRS_LDL-C_ (*P* = 5 × 10^−6^) and GRS_HDL-C_ (*P* = 0.02) but not GRS_TG_ (*P* = 0.08) were significantly associated with CVD risk. No significant interaction between the GRSs and diet quality was observed on CVD risk (*P* > 0.39). A high compared to a low diet quality attenuated the association between GRS_LDL-C_ and the risk of incident ischemic stroke (*P* interaction = 0.01).

**Conclusion:**

We found some evidence of an interaction between diet quality and GRS_LDL-C_ on ischemic stroke.

**Electronic supplementary material:**

The online version of this article (doi:10.1186/s12263-016-0536-0) contains supplementary material, which is available to authorized users.

## Background

Plasma concentrations of low-density lipoprotein-cholesterol (LDL-C) and high-density lipoprotein-cholesterol (HDL-C) are well-established prognostic factors for cardiovascular disease (CVD), in particular coronary heart disease (CHD) [12]. Lifestyle changes to reduce LDL-C and increase HDL-C, such as dietary modification and increased physical activity, are widely used in the primary prevention of CVD [29, 39]. Plasma LDL-C and triglycerides (TG) have been confirmed to be causally linked to CHD in Mendelian randomization studies [7, 15, 42]; however, so far, evidence regarding the causal relevance of HDL-C is lacking [2, 7, 15, 42]. For example, a recent Mendelian randomization study by Voight et al. found that a genetic score of 13 LDL-C-increasing alleles increased the risk of CHD, whereas a genetic score of 14 HDL-C-increasing alleles did not decrease the risk of CHD [42]. The authors concluded that some genetic mechanisms that increase HDL-C do not automatically reduce the risk of CHD. Several randomized clinical trials have evaluated the benefit of reducing LDL-C and increasing HDL-C concentrations in the tertiary prevention of CHD [2, 5]. However, none of these studies have successfully demonstrated that increasing HDL-C has a protective effect on CVD. On the other hand, studies and trials on patients with hypercholesterolemia strongly indicate that LDL-C-lowering therapies reduce the risk of CHD [1, 4], which is in line with the results from observational studies [11].

A high diet quality based on the Swedish nutrition recommendations and the Swedish dietary guidelines has been shown to reduce the risk of CVD compared to a low diet quality index [14]. The diet quality index was constructed by combining six dietary components: saturated fat, polyunsaturated fat (PUFA), fish and shellfish, dietary fiber, fruit and vegetables, and sucrose [8]. The diet quality index was associated with CVD more strongly than the individual dietary components [14], illustrating the importance of examining the whole diet with regard to disease risk. Additionally, a high diet quality was associated with decreased risk of all-cause and CVD mortality, particularly among men [9], reduced systemic inflammation [6], and lower risk of developing high TG and high LDL-C during 16 years of follow-up compared with a low diet quality [35]. However, a high diet quality index was not associated with lower incidence of type 2 diabetes in the MDC cohort [20]. We hypothesize that by taking diet quality into account, we may clarify the relationship between genetically elevated TG and LDL-C and better understand the results regarding genetically elevated HDL-C and the risk of CVD. Therefore, we examine the association between genetic susceptibility to dyslipidemia and the incidence of CVD, including coronary events and ischemic stroke, and assess whether these associations differ depending on diet quality.

## Methods

### Study population

The Malmö Diet and Cancer (MDC) study is a population-based prospective cohort including 30,447 participants, with baseline data collection conducted throughout the years 1991–1996 [3]. The study population includes individuals born during 1923–1950 [22] living in the southern part of Sweden in the third largest city, Malmö. The participants were invited via personal letters and advertisements in the local newspapers and public places. The participation rate was approximately 40 % [21], and limited Swedish language skills and mental incapacity were the only exclusion criteria. In this study, we included 28,098 participants (11,063 men and 17,035 women) with complete dietary information and anthropometric measures*.* After excluding individuals with a history of coronary events or stroke (*n* = 855), self-reported diabetes or glucose-lowering medication (*n* = 798), and successful genotyping of less than 60 % of the single-nucleotide polymorphisms (SNP) (*n* = 1,646), 24,799 participants (62 % women, age 44–74 years) remained and formed the study sample. All individuals provided written informed consent, and the ethics committee of Lund University approved the MDC study protocols (LU 51–90).

### Case definition and follow-up

In total, 3068 (1759 men and 1309 women) CVD cases were identified during 369,996 person-years of follow-up. Of these, 1814 had a coronary event and 1254 had an ischemic stroke as the first event. The mean follow-up time was 15 years (range 0–20 years). Information regarding prevalent and incident CVD was retrieved from the national Swedish Hospital Discharge register [13], the Cause-of-Death register [33], and the local stroke register in Malmö (STROMA) [17, 30, 43]. A coronary event was defined on the basis of codes 410–414 (fatal or non-fatal myocardial infarction or death due to ischemic heart disease) in the International Classification of Diseases, 9th Revision (ICD-9). Ischemic stroke was defined on the basis of code 434 (ICD-9) and diagnosed when computed tomography, magnetic resonance imaging, or autopsy could verify the infarction and/or exclude hemorrhage and non-vascular disease. A stroke was classified as unspecified if neither imaging nor autopsy was performed. Hemorrhagic or non-specific stroke cases (ICD-9 codes 430, 431, and 436) do not have the same underlying risk factors as ischemic stroke and were therefore excluded. The National Tax Board provided information on vital status and emigration. The participants contributed person-time from their date of enrollment until their first CVD event, death, emigration from Sweden, or the end of follow-up (i.e., 31 December 2010).

### Genotyping and the construction of genetic risk scores

Susceptibility to dyslipidemia was estimated by combining the validated genetic variants reported in the genome-wide association study (GWAS) meta-analysis by Teslovich et al. [38]. All SNPs (*n* = 91) that reached the GWAS significance level (i.e., *P* < 5 × 10^−8^) for TG, LDL-C, or HDL-C were genotyped except *LPA* rs1084651, *JMJD1C* rs10761731, and *NPC1L1* rs217386 because of difficulties in genotyping or a lack of proxies available. Genotyping was performed using Sequenom MassARRAY (Sequenom, San Diego, CA, USA) or Taqman allelic discrimination on an ABI 7900 (Applied Biosystems, Foster City, CA, USA) at the Clinical Research Center, Malmö, Sweden. Thereafter, the SNPs with a success rate of less than 90 % (i.e., *COBLL1* rs10195252, *KLF14* rs4731702, *PLEC1* rs11136341, and *ABCA8* rs4148008) and those with Hardy-Weinberg equilibrium *P* values less than 0.00057 (0.05/87) (i.e., *ANGPTL3* rs2131925, *TYW1B* rs13238203, *SCARB1* rs838880, *OSBPL7* rs7206971, *LILRA3* rs386000, *PLTP* rs6065906, and *MOSC1* rs2642442) were excluded. Weighted genetic risk scores (GRSs) were constructed using PLINK (version 1.07) for TG (26 SNPs), HDL-C (41 SNPs), and LDL-C (32 SNPs) (Additional file 1) by multiplying the effect size (i.e., β-coefficients) found in the meta-analysis [38] by the number of risk alleles and then summing the products. The respective GRSs explained 7.3 % of the variance in LDL-C, 5.7 % of the variance in HDL-C, and 4.7 % of the variance in TG.

### Dietary information

Dietary intake was measured by a modified diet history methodology specifically designed for the MDC study by combining a 168-item dietary questionnaire (i.e., “habitual” diet information), a 7-day menu book (i.e., “current” diet information), and a 1-h diet history interview [31, 41]. The dietary questionnaire covered food items regularly consumed during the past year. The 7-day menu book covered cooked lunch and dinner meals, cold beverages (including alcoholic beverages), medications, natural remedies, and dietary supplements used by the participants. During the interview, the menu book and questionnaire were checked for notably high reported intakes and overlapping information and the participants were asked about their food choices, food preparation practices, and portion sizes of the food reported in the menu book. The relative validity of a slightly different methodology (130-item questionnaire and 2-week food records without the interview was compared with a reference method of 18 days weighted food records with three consecutive days every second month during 1 year) in the Malmö population has been published earlier [10, 31]. The energy-adjusted correlation coefficients between the modified diet history method and the reference method were for saturated fat (0.56 and 0.68 for men and women, respectively), PUFA (0.26; 0.64), fish (0.35; 0.70), fiber (0.74; 0.69), fruit (0.60; 0.77), vegetables (0.65; 0.53), and sugar (0.60; 0.74) [10, 31].

We also noted the season of the dietary interview: winter (Jan–Mar), spring (Apr–Jun), summer (Jul–Sept), or autumn (Oct–Dec). The routines for coding dietary data were slightly altered in September 1994 to shorten the interview time, and a diet assessment method version variable was constructed to indicate whether the data were collected before or after the 1 September 1994. This change did not appear to have any major influence on the ranking of individuals [41]. The average daily intake (i.e., grams per day) of food and supplements was calculated based on the information from the menu book, interview, and questionnaire and converted into nutrient and energy intakes using the MDC Food and Nutrient Database, developed from the PC KOST-93 of the Swedish National Food Administration.

The diet quality index was developed to assess diet quality based on the Swedish nutrition recommendations issued in 2005 and has been described previously [8]. The diet quality index includes six dietary components: contribution to non-alcohol energy percentage (E%) from saturated fat, E% from PUFA, intake of fish and shellfish (g/week), dietary fiber (g/MJ), fruit and vegetables (g/day), and E% from sucrose. The following cutoffs were used: saturated fat ≤14 E%, PUFA 5-10 E%, fish and shellfish ≥300 g/week, fiber ≥2.4 g/MJ, fruit and vegetables ≥400 g/day, and sucrose ≤10 E%. The revised version of the Nordic nutrition recommendations from 2012 would not have influenced the cutoffs used. Because only 2 % of the participants had an intake below the recommended level (≤10 E%) for saturated fat, the cutoff for saturated fat was increased to ≤14 E% (i.e., one standard deviation (SD) increase). One point was given to the participants for each dietary component that reached the recommended intake level, and zero points were given if they were not within the recommended range. A total score was created by summing the points and divided into three categories: low (0–1 point), medium (2–4 points), and high (5–6 points).

Information regarding dietary change in the past (yes/no) was based on the question “Have you substantially changed your eating habits because of illness or for other reasons?”. The dietary habits of participants reporting dietary changes may reflect only a short period of their lives and may therefore have less influence on the development of chronic disease. Potential misreports of energy intake were identified by comparing reported energy intake with total energy expenditure (i.e., estimated from a calculated basal metabolic rate and self-reports of leisure time physical activity, work activity, household work, and sleep hours). Individuals with reported energy intake outside the 95 % confidence interval (CI) for total energy expenditure were categorized as “misreporters”, as is further explained elsewhere [23].

### Other variables

Age and sex were identified from each individual’s civil registration number. Body mass index (BMI) (kg/m^2^) was calculated from a direct measurement of weight and height, wearing light clothes and no shoes, conducted by a nurse. A self-administered questionnaire was used to determine lifestyle and socioeconomic factors including smoking habits, alcohol habits, physical activity habits, and educational level, current medication, diet supplement use, and history of diseases. Three categories of smoking habits were used: current (including irregular smoking), former, and never smokers. Individuals were divided into five categories based on their alcohol habits. Individuals reporting no alcohol consumption during the last year in the questionnaire, who were also zero reporters of alcohol in the 7-day menu book, were categorized as zero consumers of alcohol. We divided the other study participants into categories (low, moderate, high, and very high) based on their alcohol consumption (grams per day) with different cutoffs according to gender. The cutoff levels for females were 5, 10, and 20 g of alcohol per day, and the cutoff levels for males were 10, 20, and 40 g of alcohol per day. Educational level was categorized based on the highest level of education attained: elementary or less, primary and secondary, upper secondary, further education without a degree, and university degree. Physical activity levels during leisure time were calculated from a list of 17 different activities in the questionnaire. The time spent on each activity was multiplied with an intensity factor, creating a leisure time physical activity score [19]. The leisure time physical activity score was then divided into quintiles with the same cutoffs for both genders because of similar scores in men and women. Separate categories for smoking habits, alcohol habits, educational level, and leisure time physical activity were constructed for the participants with missing data.

### Statistical analyses

Statistical Package of the Social Science (SPSS, version 22.0; IBM Corporation, Armonk, NY, USA) was used for statistical analyses. Differences in baseline characteristics across the diet quality categories (i.e., low, medium, and high) and GRSs per SD were tested in men and women separately using chi^2^-test for categorical variables and general linear model adjusted for age for continuous variables. All continuous variables except age were logarithmically (Ln) transformed to achieve a normal distribution when testing for trends across the diet quality index (0–6) and GRSs (continuous); before transformation, a very small amount (i.e., 0.001 g) was added to fish and shellfish and fruit and vegetables, intakes to handle zero intakes. Additionally, differences in the number of diet changers and non-diet changers between tertiles of GRSs were tested using chi^2^-test. Linear regression was used to examine the association between the GRSs and TG and HDL-C and LDL-C concentrations at baseline. For analyses of the *P* for trend and variance explained, we used Ln-transformed TG, LDL-C, and HDL-C. Cox proportional hazard regression was used to examine associations between GRSs and incident CVD, coronary events, and ischemic stroke, adjusted for age and sex and with years of follow-up as the underlying time variable. We also added dietary fiber in this model because it is a known risk factor for CVD and for being associated with all three GRSs in this study. Additionally, we examined these associations in strata of diet quality categories (i.e., low, medium, and high) adjusted for age and sex and both with and without BMI because BMI can be regarded as a mediating factor. The combined diet index was the main dietary variable, but we also examined the associations between the GRSs and CVD with adherence to each component separately. The interactions between the diet quality index and the three GRSs on CVD were examined by introducing multiplicative factors of GRSs and diet quality index as continuous variables, in addition to the main factors as separate variables, in a multivariable model adjusted for age, sex, dietary assessment method version, season, total energy intake, smoking habits, alcohol habits, leisure time physical activity, educational level, and BMI. These variables were selected from the literature for being known risk factors for CVD [26, 29] and for being associated with dietary intakes in this study. Because many of the SNPs included in the GRSs have pleiotropic effects, the analyses for each GRS were adjusted for the other two GRSs. To further interpret the statistically significant interactions between GRSs and the diet quality index on CVD risk, we examined associations with diet quality index (both as a continuous and categorical variable) in tertiles of GRSs. The lowest diet quality index category was used as the reference. All analyses were also carried out separately in men and women due to gender differences in food selection and reporting, as well as biological differences. Formal tests for interactions by sex were also performed. In sensitivity analyses, we excluded individuals reporting dietary changes in the past, because they may have unstable food habits, and potential “misreporters” of energy intake.

## Results

### Baseline characteristics

Baseline characteristics across the diet quality categories are shown in Additional file 2. GRSs were composed of 26 SNPs for TG, 32 SNPs for LDL-C, and 41 SNPs for HDL-C (Additional file 1). The GRSs explained 4.7, 7.3, and 5.7 %, respectively, of the variance in the traits (Additional file 3). We observed several statistically significant associations between baseline characteristics and the GRSs; for example, all the GRSs were associated with higher dietary fiber intakes and lower saturated fat intakes (Additional file 4). These associations were in the same direction in men and women. In addition, we observed a significantly higher frequency of diet changers in the highest tertile of GRS_LDL-C_ compared to the lowest tertile (23 and 20 % *P* (chi^2^-test) = 6 × 10^−5^). Similar tendencies were observed for GRS_HDL-C_ (*P* = 0.06) and GRS_TG_ (*P* = 0.09) (Additional file 4). The associations between dietary intakes and GRSs were attenuated when excluding diet changers in the past and potential misreporters of energy.

### Genetic risk for dyslipidemia and CVD

GRS_LDL-C_ was significantly associated with an increased risk of CVD (*P* = 5 × 10^−6^), coronary events (*P* = 1.5 × 10^−4^), and ischemic stroke (*P* = 0.02) after adjusting for age and sex (Table 1). GRS_HDL-C_ was significantly associated with an increased risk of CVD (*P* = 0.02) and ischemic stroke (*P* = 0.04) but not with an increased risk of coronary events (*P* = 0.18). GRS_TG_ was significantly associated with an increased risk of coronary events (*P* = 0.01) but not with an increased risk of CVD (*P* = 0.08) and ischemic stroke (*P* = 0.59).

**Table 1:** HR of incident CVD, coronary event, and ischemic stroke per 1 SD increase of GRS

GRSs	Total CVD		Coronary event		Ischemic stroke	
	Cases *n*_men_ = 1759/*n*_women_ = 1309		Cases *n*_men_ = 1129/*n*_women_ = 685		Cases *n*_men_ = 630/*n*_women_ = 624	
	Model 1^a^	Model 2^b^	Model 1	Model 2	Model 1	Model 2
GRS_LDL-C_						
All	1.09 (1.05–1.13)	1.08 (1.04–1.12)	1.09 (1.04–1.15)	1.08 (1.03–1.14)	1.07 (1.01–1.13)	1.07 (1.01–1.14)
Men	1.08 (1.03–1.13)	1.07 (1.02–1.12)	1.08 (1.02–1.14)	1.07 (1.01–1.14)	1.07 (0.99–1.16)	1.07 (0.99–1.16)
Women	1.10 (1.04–1.16)	1.09 (1.03–1.15)	1.13 (1.05–1.22)	1.11 (1.03–1.20)	1.07 (0.99–1.16)	1.07 (0.99–1.16)
GRS_HDL-C_						
All	1.05 (1.01–1.08)	1.03 (1.00–1.07)	1.03 (0.99–1.08)	1.00 (0.95–1.05)	1.06 (1.00–1.12)	1.08 (1.01–1.14)
Men	1.03 (0.98–1.08)	1.01 (0.96–1.06)	1.01 (0.95–1.07)	0.97 (0.91–1.04)	1.06 (0.98–1.15)	1.08 (0.99–1.18)
Women	1.07 (1.01–1.13)	1.06 (1.00–1.13)	1.08 (1.00–1.16)	1.05 (0.97–1.14)	1.06 (0.98–1.14)	1.07 (0.98–1.17)
GRS_TG_						
All	1.03 (1.00–1.07)	1.00 (0.96–1.04)	1.07 (1.02–1.12)	1.05 (1.00–1.10)	0.99 (0.93–1.04)	0.94 (0.88–1.00)
Men	1.03 (0.99–1.08)	1.02 (0.96–1.07)	1.06 (1.00–1.13)	1.06 (1.00–1.14)	0.98 (0.91–1.06)	0.94 (0.86–1.02)
Women	1.03 (0.98–1.09)	0.98 (0.93–1.04)	1.07 (0.99–1.15)	1.02 (0.94–1.11)	0.99 (0.91–1.07)	0.94 (0.86–1.03)

Cox proportional hazard regression model was used to calculate HRs (95 % CI), among 9383 men and 15,416 women in the Malmö Diet and Cancer cohort

^a^Model 1 is adjusted for age and sex

^b^Model 2 is adjusted for age, sex, and the two GRSs simultaneously

When we adjusted for pleiotrophy (i.e., adding the other two GRSs to the statistical model), the estimated risk was slightly changed, especially the associations with GRS_HDL-C_ and GRS_TG_ (Table 1). Adding dietary fiber to this model did not markedly change the results (data not shown).

### Interaction with diet quality index

**Fig. 1:**
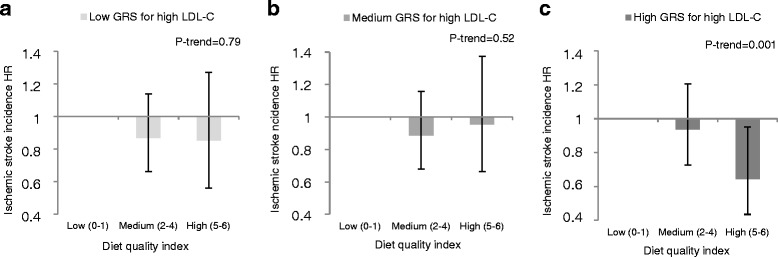
Association between the diet quality index and incidence of ischemic stroke according to tertiles of GRS_LDL-C_, low (**a**), medium (**b**) and high (**c**) among 24,799 participants in the Malmö Diet and Cancer cohort. A Cox proportional hazard regression was used to calculate HR for each diet quality category with the lowest category as a reference. Multivariable models were adjusted for age, sex, BMI, diet assessment method version, season, total energy intake, alcohol habits, leisure time physical activity, educational level, and smoking habits. In tertiles of GRS_LDL-C_ (non-cases/cases) of ischemic stroke; low *n* = 7293/395; medium *n* = 7272/415; high *n* = 7166/444

No significant interaction between the GRSs and diet quality was observed for the incidence of CVD (GRS_LDL-C_*P* = 0.39, GRS_HDL-C_*P* = 0.85, and GRS_TG_*P* = 0.86) (Table 2). When coronary and ischemic stroke events were examined separately, we observed a significant interaction between GRS_LDL-C_ and diet quality index on ischemic stroke incidence (*P* = 0.01). When the analysis was stratified by diet quality categories, a high compared to a low diet quality attenuated the association between GRS_LDL-C_ and the increased risk of incident ischemic stroke (hazard ratio (HR) per SD, HR_low_ = 1.08 (0.95–1.24), *P =* 0.26; HR_medium_ = 1.10 (1.03–1.17), *P =* 0.01; HR_high_ = 0.93 (0.79–1.10), *P =* 0.40 (Table 2)). This association became significant when we combined the low and medium diet quality group (HR_low+medium_ = 1.09; 95 % CI, 1.03–1.16; *P* = 0.004). When we examined the association between the diet quality index and the incidence of ischemic stroke in tertiles of GRS_LDL-C_, we observed only a significant inverse association between the diet quality index and the incidence of ischemic stroke among the participants in the highest tertile of GRS_LDL-C_ (HR for highest vs. lowest diet quality = 0.64 (0.44–0.95), *P* trend = 0.001) (Fig. 1). Adding BMI to the statistical model did not markedly change the results. Thereafter, we examined the interaction between the GRSs and each diet index component on the incidence of CVD, coronary events, and ischemic stroke. There was no significant interaction between any of the diet index components and the GRS_LDL-C_ (*P*_lowest for PUFA_ = 0.22 to *P*_highest for fish and shellfish_ = 0.95), GRS_HDL-C_ (*P*_lowest for fish and shellfish_ = 0.18 to *P*_highest for PUFA_ = 0.81), and GRS_TG_ (*P*_lowest for sucrose_ = 0.09 to *P*_highest for PUFA_ = 0.96) on the incidence of CVD (Additional file 5). However, we observed a significant interaction between GRS_LDL-C_ and fruit and vegetable intake (*P* = 0.01) on ischemic stroke incidence. The association between GRS_LDL-C_ and increased risk of ischemic stroke was attenuated among the participants with high intake of fruit and vegetables (>400 g/day). No significant interactions were observed between the GRSs and diet quality index components on the incidence of coronary events (Additional file 5).

**Table 2:** HR in strata of diet quality index on incident CVD, coronary event, and ischemic stroke

	Diet quality index			*P* interaction^a^
	Low	Medium	High	
	*n* = 3360	*n* = 15,538	*n* = 2833	
	HR (95 % CI)	HR (95 % CI)	HR (95 % CI)	
Total CVD	530 cases	2186 cases	352 cases	
GRS_LDL-C_	1.11 (1.02–1.21)	1.09 (1.04–1.14)	1.07 (0.96–1.19)	0.39 (0.86)^b^
GRS_HDL-C_	1.08 (0.99–1.18)	1.03 (0.99–1.07)	1.10 (0.99–1.22)	0.85 (0.58)
GRS_TG_	1.02 (0.93–1.11)	1.03 (0.99–1.08)	1.05 (0.95–1.17)	0.86 (0.20)
Coronary event	Cases *n* = 313	Cases *n* = 1285	Cases *n* = 216	
GRS_LDL-C_	1.13 (1.01–1.26)	1.08 (1.02–1.14)	1.15 (1.01–1.32)	0.33 (0.08)
GRS_HDL-C_	1.02 (0.91–1.14)	1.03 (0.97–1.08)	1.11 (0.97–1.27)	0.35 (0.78)
GRS_TG_	1.06 (0.95–1.19)	1.06 (1.01–1.12)	1.09 (0.95–1.25)	0.78 (0.23)
Ischemic stroke	Cases *n* = 217	Cases *n* = 901	Cases *n* = 136	
GRS_LDL-C_	1.08 (0.95–1.24)	1.10 (1.03–1.17)	0.93 (0.79–1.10)	0.01 (0.07)
GRS_HDL-C_	1.16 (1.02–1.33)	1.04 (0.97–1.11)	1.07 (0.91–1.26)	0.18 (0.21)
GRS_TG_	0.96 (0.84–1.10)	0.99 (0.93–1.06)	0.99 (0.83–1.17)	0.98 (0.59)

Cox proportional hazard regression was used to calculate HRs (95 % CI) per 1 SD increase of the GRSs, *P* < 0.05, adjusted for age and sex among 24,799 participants in the Malmö Diet and Cancer cohort

^a^ *P* interactions (GRSs × diet quality index as continuous variables) adjusted for age, sex, BMI, diet assessment method version, season, total energy intake, alcohol habits, leisure time physical activity, educational level, and smoking habits

^b^ *P* values in parentheses are sensitivity analyses excluding those reporting dietary changes in the past and potential energy misreporters, *n* = 16,030

Furthermore, we preformed all analyses separately in men and women. There were no three-way statistical interactions (i.e., sex*diet quality index*GRS) on CVD (*P* > 0.48), coronary events (*P* > 0.36), or ischemic stroke (*P* > 0.38). However, the interaction between GRS_LDL-C_ and the diet quality index on ischemic stroke was statistically significant in women (*P* = 0.03) but not in men (*P* = 0.12) (Additional file 6). This interaction was mainly driven by fruit and vegetable intakes (*P* = 1 × 10^−3^) in men and saturated fat (*P* = 0.02) and PUFA (*P* = 0.06) in women (Additional file 5). There was only a significant association between GRS_LDL-C_ and increased risk of ischemic stroke among men with low fruit and vegetable intake (≤400 g/day). In women, a significant association between GRS_LDL-C_ and increased risk of ischemic stroke was observed only among those with high intake of saturated fat (≥14 E%) and outside the recommended range of PUFA (5–10 E%).

In sensitivity analyses, we excluded participants reporting dietary changes in the past and those with suspected misreporting of energy (*n* = 8,769; 35 % of the study sample). In line with the results reported above, no significant interactions between the GRSs and diet quality index on the incidence of CVD were observed (GRS_LDL-C_*P* = 0.86, GRS_HDL-C_*P* = 0.58, and GRS_TG_*P* = 0.20). The interaction between GRS_LDL-C_ and the diet quality index on ischemic stroke was somewhat attenuated in this reduced study sample (*P* = 0.07). The interaction between GRS_LDL-C_ and fruit and vegetable intake on ischemic stroke remained statistically significant (*P* = 0.049). Overall, the results did not markedly change when adding the two GRSs simultaneously to the statistical model.

## Discussion

In this study, we examined the association between genetic susceptibility to dyslipidemia and the risk of CVD by combining 80 validated genetic variants associated with blood lipids and lipoproteins. These variants have been suggested to account for approximately 25–30 % of the genetic variance of blood lipid and lipoprotein concentrations [38] and can therefore be used as a marker of genetic susceptibility to dyslipidemia in Caucasians.

We found some evidence of an interaction between diet quality and GRS_LDL-C_ affecting the risk of ischemic stroke but not CVD or coronary events. A high compared to a low diet quality attenuated the genetic susceptibility of high LDL-C on the risk of ischemic stroke incidence.

The GRS_LDL-C_ was significantly associated with an increased risk of CVD and coronary events. This is in line with a number of Mendelian randomization studies [7, 15, 42]. Additionally, numerous potential genetic risk alleles for stroke have been reported; however, the evidence is so far not conclusive [16], and studies examining the association between GRS_LDL-C_ and stroke are missing. In the present study, a high compared to low diet quality attenuated the association between GRS_LDL-C_ and the increased risk of incident ischemic stroke. Although GRS_LDL-C_ tended to be associated with increased risk in the low and medium diet quality groups, no such tendency was observed in the high diet quality group. However, the non-significant trend among the participants in the high and low diet quality groups might, at least in part, be explained by a lower number of individuals in these groups (*n*_low_ = 3890, *n*_medium_ = 17,724, and *n*_high_ = 3185). When we divided the GRS_LDL-C_ into tertiles, we observed that a high diet quality was inversely associated with ischemic stroke incidence among individuals in the highest tertile of GRS_LDL-C_. The observed interaction between GRS_LDL-C_ and the diet quality index on ischemic stroke seems to mainly be driven by fruit and vegetable intakes in men and fat quality (i.e., saturated fat and PUFA) in women, although no significant heterogeneities regarding these interactions were observed between the sexes.

The included genetic variants are located in or near genes that are involved in various and, in many cases, unknown mechanisms and pathways of lipid and lipoprotein metabolism, which may attenuate the interactions when they are combined into GRSs. It may therefore be important to examine genetic variants affecting specific mechanisms and pathways separately to address whether genetic susceptibility affecting such specific mechanisms or pathways would modify the associations. In addition, several of the SNPs included in the GRSs have pleiotropic effects associated with several lipid traits, which could bias our results. We chose to include these SNPs to avoid weakening the effects of the GRSs and corrected for pleiotropic associations in the statistical models. In addition, it is important to note the small overlap between variants identified in GWAS for cardio-metabolic traits and variants showing indication of gene-environment interaction [27]. Genetic variants under genetic pressure may be more prone to interaction with environmental factors [28].

The GRSs are composed of common genetic variants (<0.05 minor allele frequency). Low-frequency missense variants have been associated with larger differences in lipid and lipoprotein concentrations and with coronary artery disease [4, 18, 24, 32]. For example, carriers of loss-of-function mutations in *ANGPTL4* had 35 % lower TG concentrations than non-carriers; these mutations were also associated with protection from coronary artery disease [24]. Since these low-frequency variants markedly affect TG concentrations, they may be useful to include when examining interactions with diet on CVD incidence; however to conduct these kind of studies, the study material has to be extremely large.

In this type of population-based studies, the participants are often healthier than the general population. This may contribute to fewer cases of CVD and also narrow the range of dietary intakes among the participants. This may decrease the likelihood of observing differences in genetic risk for dyslipidemia and CVD depending on diet quality. The significant associations between several baseline characteristics, (e.g., higher dietary fiber and lower saturated fat intakes) and high GRSs, are not easy to explain. It may, however, indicate that those individuals with high genetic risk for dyslipidemia are more likely to be aware of their dietary habits. An explanation may be that these individuals have experienced different health problems previously and may therefore have changed their food habits. This hypothesis is supported by our observation of significantly higher frequency of diet changers in the highest tertile of GRS_LDL-C_ compared to the lowest tertile. Similar tendencies were observed for GRS_HDL-C_ and GRS_TG_. However, the associations between these baseline characteristics and GRSs were in the same direction, but attenuated, when excluding diet changers in the past and potential misreporters of energy. Unfortunately, we have dietary data only from the baseline examination, and therefore, we do not have any information regarding changes of exposure that might have occurred during the 15 years of follow-up. However, diet changers are most likely prone to unstable food habits [34, 37]. In addition, studies on long-term reproducibility have indicated that single measurements of dietary intakes can be used as proxies of long-term exposures [25, 40], and because the participants in the MDC study were middle-aged at the baseline examinations during the 90s, they are more likely to have well-established food habits than a younger population.

The strengths of this study are the prospective design, large sample size, and detailed information on dietary intakes based on a 168-item dietary questionnaire, a 7-day record, and a 1-h interview. Other major strengths of this study are the almost complete follow-up of participants through registers and the detailed ascertainment and verification of CVD diagnosis [13, 17, 30, 43].

The dietary assessment method used in the MDC study was specifically designed to measure intakes of vegetables, fiber, and fat [3], and the relative validity of the method is generally high. The high validity may contribute to the ability to observe significant interactions between GRS_LDL-C_ and fruit and vegetable intakes affecting ischemic stroke incidence. However, the relative validity is rather low for fish and PUFA intake in men. Because fish consumed at main meals was registered only during seven consecutive days and fish is likely to be consumed relatively infrequently, misclassification might be a problem. The high relative validity of PUFA intakes in women (0.64) compared to men (0.26) might explain why we observed a nominal interaction between GRS_LDL-C_ and PUFA intake affecting ischemic stroke in women but not in men.

The diet quality index itself could be a limitation and is only one of several ways to examine associations between diet quality and disease risk. The diet quality index has previously been shown to reflect overall diet quality and sufficiently rank participants in the MDC cohort into low, medium, and high diet quality on the basis of their intake of a wide range of foods and nutrients and may thus be more predictive of disease risk than individual foods or nutrients. However, reducing dietary habits, which are very complex, into a diet quality index constructed by adding a few dichotomous diet variables may be a disadvantage. Additionally, there may be individual dietary components that interact more strongly with GRSs [35, 36]. Dietary patterns are likely to vary according to social and cultural backgrounds; therefore, it is necessary to replicate our results in other populations. Although we adjusted for potential confounders, the diet quality index might correlate with other factors important for CVD risk; thus, residual confounding can still occur.

## Conclusions

In conclusion, we found no convincing evidence that dietary quality modifies the association between the GRSs for TG, LDL-C, and HDL-C and CVD risk. Further studies may need to consider different mechanisms and pathways for genetic variants associated with dyslipidemia separately to clarify the interaction between GRSs and diet quality on CVD risk. Finally, we performed multiple tests; thus, some of the observed significant associations and interactions could be due to chance and need to be replicated. In addition, to examine the modifying effect of diet quality, further studies are needed to examine whether any specific dietary factors may modify the associations between genetic susceptibility to dyslipidemia and the incidence of CVD.

## Abbreviations

BMI, body mass index; CHD, coronary heart disease; CI, confidence interval; CVD, cardiovascular disease; E%, energy percentage; GRS, genetic risk score; GWAS, genome-wide association study; HR, hazard ratio; ICD-9, International Classification of Diseases, 9th revision; MAF, minor allele frequency; MDC, Malmö Diet and Cancer; PUFA, polyunsaturated fat; SD, standard deviation; SE, standard error; SNP, single-nucleotide polymorphism; STROMA, stroke register in Malmö

## Additional files

Additional file 1: Characteristics of the included single nucleotide polymorphisms. (DOCX 21 kb) Additional file 2: Baseline characteristics among 9383 men and 15,416 women in the Malmö Diet and Cancer cohort according to the diet quality index. (DOCX 22 kb) Additional file 3: Association between the genetic risk scores and LDL-C, HDL-C, and TG in the Malmö Diet and Cancer cohort. (DOCX 17 kb) Additional file 4: Association (*P* values and directions) between baseline characteristics and the genetic risk scores among 24,799 participants in the Malmö Diet and Cancer cohort. (DOCX 19 kb) Additional file 5: Interaction (*P* value) between the genetic risk scores and the diet index components on incidence of total cardiovascular disease, coronary event, and ischemic stroke among 24,799 participants in the Malmö Diet and Cancer cohort. (DOCX 20 kb) Additional file 6: Hazard ratios for per 1 SD increase of the genetic risk scores in strata of diet quality index on incidence of total cardiovascular disease, coronary event, and ischemic stroke among 9383 men and 15,416 women in the Malmö Diet and Cancer cohort. (DOCX 19 kb)
