# Supplementary material for: Genetic susceptibility to dyslipidemia and incidence of cardiovascular disease depending on a diet quality index in the Malmö Diet and Cancer cohort
Source: Genes Nutr. 2016 Jul 7;11:20. doi: 10.1186/s12263-016-0536-0 (PMC4968442; doi:10.1186/s12263-016-0536-0)
Supplement: Additional file 2: — Baseline characteristics among 9383 men and 15,416 women in the Malmö Diet and Cancer cohort according to the diet quality index. (DOCX 22 kb) [file 12263_2016_536_MOESM2_ESM.docx]

**Additional file 2:**

**Title:** Genetics susceptibility to dyslipidemia and incidence of cardiovascular disease depending on a diet quality index in the Malmö Diet and Cancer cohort.

**Journal name**: Genes and Nutrition

**Authors**: Sophie Hellstrand, Ulrika Ericson, Christina-Alexandra Schulz, Isabel Drake, Bo Gullberg, Bo Hedblad, Gunnar Engström, Marju Orho-Melander, Emily Sonestedt

**Affiliation**: Diabetes and Cardiovascular Disease – Genetic Epidemiology, Department of Clinical Sciences in Malmö, Lund University, Sweden

**Corresponding author**: sophie.hellstrand@med.lu.se

**Additional file 2**. Baseline characteristics among 9,383 men and 15,416 women in the Malmö Diet and Cancer cohort according to the diet quality index^1^

| Characteristics | Men | | | | Women | | | |
| --- | --- | --- | --- | --- | --- | --- | --- | --- |
|  | Diet quality index | | |  | Diet quality index | | |  |
|  | Low  (0-1) | Medium  (2-4) | High  (5-6) | *P-*trend^3^ | Low  (0-1) | Medium  (2-4) | High  (5-6) | *P-*trend^3^ |
| Number of participants^2^ | 1404 | 6991 | 988 |  | 2486 | 10 733 | 2197 |  |
| Age, y^3^ | 59.0 (58.6-59.4) | 58.9 (58.7-59.1) | 59.5 (59.0-59.9) | 0.11 | 57.0 (56.7-57.4) | 57.2 (57.0-57.3) | 57.6 (57.3-58.0) | <0.001 |
| BMI, kg/m | 25.8 (25.6-26.0) | 26.2 (26.1-26.3) | 26.3 (26.1-26.5) | <0.001 | 25.0 (24.8-25.1) | 25.4 (25.3-25.4) | 25.6 (25.4-25.7) | <0.001 |
| LDL-C^4^ | 4.10 (3.5-4.6) | 4.10 (3.5-4.7) | 4.10 (3.5-4.8) | 0.39 | 4.10 (3.5-4.9) | 4.10 (3.4-4.8) | 4.20 (3.5-4.9) | 0.09 |
| HDL-C | 1.11 (0.9-1.3) | 1.19 (1.0-1.4) | 1.18 (1.0-1.4) | 0.01 | 1.43 (1.2-1.7) | 1.47 (1.3-1.7) | 1.48 (1.3-1.8) | 0.01 |
| TG | 1.39 (1.0-1.9) | 1.24 (0.9-1.7) | 1.27 (0.9-1.7) | 0.32 | 1.13 (0.8-1.5) | 1.08 (0.8-1.5) | 1.03 (0.8-1.4) | 0.11 |
| Dietary intakes |  |  |  |  |  |  |  |  |
| Total energy intake, *MJ/day* | 11.2 (11.0-11.3) | 11.2 (11.1-11.3) | 10.9 (10.7-11.0) | 0.12 | 8.7 (8.6-8.8) | 8.5 (8.5-8.6) | 8.3 (8.2-8.4) | <0.001 |
| Saturated fat, *E%* | 18.4 (18.2-18.5) | 16.6 (16.5-16.7) | 12.6 (12.4-12.9) | <0.001 | 18.1 (18.0-18.3) | 16.3 (16.2-16.4) | 13.0 (12.8-13.1) | <0.001 |
| PUFA, *E%* | 5.2 (5.1-5.3) | 6.3 (6.3-6.4) | 6.3 (6.2-6.4) | <0.001 | 5.1 (5.0-5.1) | 5.9 (5.8-5.9) | 6.1 (6.1-6.2) | <0.001 |
| Fish and shellfish, *g/week* | 174.2 (160.2-188.3) | 362.1 (355.8-368.4) | 522.0 (505.3-538.8) | <0.001 | 172.2 (164.4-180.1) | 296.7 (293.0-300.4) | 449.1 (440.8-457.4) | <0.001 |
| Dietary fiber, *g/MJ* | 1.7 (1.6-1.7) | 2.0 (2.0-2.0) | 2.9 (2.8-2.9) | <0.001 | 1.8 (1.8-1.8) | 2.3 (2.3-2.3) | 3.0 (3.0-3.0) | <0.001 |
| Fruit and vegetables, *g/day* | 241.5 (233.0-250.0) | 336.3 (332.5-340.1) | 549.9 (539.8-560.0) | <0.001 | 271.7 (265.2-278.1) | 387.5 (384.4-390.6) | 568.3 (561.5-575.2) | <0.001 |
| Sucrose, *E%* | 11.3 (11.1-11.5) | 7.7 (7.6-7.8) | 6.9 (6.7-7.1) | <0.001 | 11.3 (11.2-11.4) | 8.5 (8.4-8.6) | 7.6 (7.5-7.7) | <0.001 |
| Alcohol habits, % |  |  |  | <0.001 |  |  |  | <0.001 |
| Zero reporters | 6.5 | 3.9 | 3.8 |  | 10.7 | 6.8 | 5.9 |  |
| Low | 47.3 | 38.7 | 42.7 |  | 45.5 | 39.0 | 40.9 |  |
| Moderate | 22.2 | 25.7 | 28.8 |  | 19.0 | 23.0 | 23.3 |  |
| High | 18.2 | 23.7 | 19.7 |  | 17.5 | 22.2 | 21.9 |  |
| Very high | 5.8 | 8.0 | 4.9 |  | 7.4 | 9.1 | 8.0 |  |
| Smoking status, *%* |  |  |  | <0.001 |  |  |  | <0.001 |
| Current smoker | 38.3 | 28.6 | 15.0 |  | 37.4 | 27.8 | 17.2 |  |
| Ex-smoker | 35.6 | 42.5 | 48.8 |  | 23.1 | 27.5 | 34.0 |  |
| Never smoker | 26.1 | 28.8 | 36.2 |  | 39.5 | 44.7 | 48.8 |  |
| Educational level, *%* |  |  |  | <0.001 |  |  |  | <0.001 |
| Elementary or less | 53.0 | 45.0 | 37.4 |  | 43.9 | 38.4 | 35.2 |  |
| Primary and secondary | 18.6 | 19.7 | 22.0 |  | 29.2 | 30.8 | 31.0 |  |
| Upper secondary | 9.2 | 12.3 | 13,6 |  | 7.4 | 7.0 | 6.7 |  |
| Further education without a degree | 7.6 | 9.4 | 11.8 |  | 7.6 | 8.3 | 10.5 |  |
| University degree | 11.6 | 13.5 | 15.2 |  | 11.9 | 15.5 | 16.5 |  |
| Leisure time physical activity, *%* |  |  |  | <0.001 |  |  |  | <0.001 |
| Very low | 15.9 | 10.3 | 5.7 |  | 12.7 | 9.2 | 5.6 |  |
| Low | 35.6 | 32.4 | 25.0 |  | 38.0 | 33.9 | 29.6 |  |
| Medium | 22.7 | 28.1 | 30.3 |  | 26.1 | 29.6 | 30.9 |  |
| High | 16.9 | 19.5 | 24.5 |  | 16.0 | 19.4 | 23.4 |  |
| Very high | 8.9 | 9.8 | 14.6 |  | 7.2 | 7.9 | 10.5 |  |

^1^The diet quality index ranged from 0 to 6 according to the adherence to the dietary components. Data is presented as means (95% CI) for continuous variables and as proportions (%) for categorical variables,

if not otherwise indicated.

^2^Number of participants: BMI (n=24,770); educational level (n=24,743); leisure time physical activity (n=24,647); and smoking habits (n=24,789).

^3^Differences in baseline characteristics between diet quality categories using the General Linear Model for continuous variables and Chi^2^ test for categorical variables (unadjusted), *P* < 0.05. *P*-trends are presented

for the continuous variables with the diet quality index as a continuous variable adjusted for age and Ln-transformed dependent variables (except age).

^4^LDL-C (n=4,926), HDL-C (n=4,864) and TG (n=4,974) are presented as medians (inter-quartile range). Abbreviation: E%, energy percentage.
