# Supplementary material for: Genetic susceptibility to dyslipidemia and incidence of cardiovascular disease depending on a diet quality index in the Malmö Diet and Cancer cohort
Source: Genes Nutr. 2016 Jul 7;11:20. doi: 10.1186/s12263-016-0536-0 (PMC4968442; doi:10.1186/s12263-016-0536-0)
Supplement: Additional file 3: — Association between the genetic risk scores and LDL-C, HDL-C, and TG in the Malmö Diet and Cancer cohort. (DOCX 17 kb) [file 12263_2016_536_MOESM3_ESM.docx]

**Additional file 3:**

**Title:** Genetics susceptibility to dyslipidemia and incidence of cardiovascular disease depending on a diet quality index in the Malmö Diet and Cancer cohort.

**Journal name**: Genes and Nutrition

**Authors**: Sophie Hellstrand, Ulrika Ericson, Christina-Alexandra Schulz, Isabel Drake, Bo Gullberg, Bo Hedblad, Gunnar Engström, Marju Orho-Melander, Emily Sonestedt

**Affiliation**: Diabetes and Cardiovascular Disease – Genetic Epidemiology, Department of Clinical Sciences in Malmö, Lund University, Sweden

**Corresponding author**: sophie.hellstrand@med.lu.se

**Additional file 3.** Association between the genetic risk scores and LDL-C, HDL-C and TG in the Malmö Diet and Cancer cohort^1^

|  | | GRS_LDL-C_ | |  | GRS_HDL-C_ | |  | GRS_TG_ | |  |
| --- | --- | --- | --- | --- | --- | --- | --- | --- | --- | --- |
|  | β^2^  mmol/L | | *P* | Variance explained (%) | β^2^  mmol/L | *P* | Variance explained (%) | β^2^  mmol/L | *P* | Variance explained (%) |
| LDL-C | 0.272 (0.014) | | 2 x 10^-82^ | 7.3 | 0.039 (0.014) | 0.002 | 0.2 | 0.091 (0.014) | 2 x 10^-10^ | 0.8 |
| HDL-C | -0.014 (0.005) | | 0.005 | 0.1 | -0.090 (0.005) | 2 x 10^-65^ | 5.7 | -0.043 (0.005) | 4 x 10^-17^ | 1.4 |
| TG | 0.054 (0.011) | | 7 x 10^-8^ | 0.6 | 0.070 (0.011) | 3 x 10^-11^ | 0.9 | 0.151 (0.010) | 1 x 10^-53^ | 4.7 |

^1^The linear regression model, Ln-transformed LDL-C, HDL-C and TG.

^2^β = Effect size (SE) per 1 SD increase of GRS on each trait, not Ln-transformed. Abbreviations: GRS, genetic risk scores; SD, standard deviation; SE, standard error.
