# Supplementary material for: Genetic susceptibility to dyslipidemia and incidence of cardiovascular disease depending on a diet quality index in the Malmö Diet and Cancer cohort
Source: Genes Nutr. 2016 Jul 7;11:20. doi: 10.1186/s12263-016-0536-0 (PMC4968442; doi:10.1186/s12263-016-0536-0)
Supplement: Additional file 4: — Association (P values and directions) between baseline characteristics and the genetic risk scores among 24,799 participants in the Malmö Diet and Cancer cohort. (DOCX 19 kb) [file 12263_2016_536_MOESM4_ESM.docx]

**Additional file 4:**

**Title:** Genetics susceptibility to dyslipidemia and incidence of cardiovascular disease depending on a diet quality index in the Malmö Diet and Cancer cohort.

**Journal name**: Genes and Nutrition

**Authors**: Sophie Hellstrand, Ulrika Ericson, Christina-Alexandra Schulz, Isabel Drake, Bo Gullberg, Bo Hedblad, Gunnar Engström, Marju Orho-Melander, Emily Sonestedt

**Affiliation**: Diabetes and Cardiovascular Disease – Genetic Epidemiology, Department of Clinical Sciences in Malmö, Lund University, Sweden

**Corresponding author**: sophie.hellstrand@med.lu.se

**Additional file 4**. Association (*P*-values and directions) between baseline characteristics and the

genetic risk scores among 24,799 participants in the Malmö Diet and Cancer cohort^1^

| Characteristics | GRS_LDL-C_ | GRS_HDL-C_ | GRS_TG_ |  |
| --- | --- | --- | --- | --- |
|  |  | *P*-trend |  |  |
| Age | 0.02(-)^2^ | 0.01(-) | 4x10^-3^(-) |  |
| BMI^3^ | 0.09(-) | 0.12(-) | 0.53(-) |  |
| Dietary intakes |  |  |  |  |
| Total energy intake  Diet quality index | 0.24(-)  1x10^-5^(+) | 4x10^-3^(-)  0.43(+) | 5x10^-3^(-)  0.54(+) |  |
| Saturated fat | 1x10^-6^(-) | 0.03(-) | 4x10^-3^(-) |  |
| PUFA | 0.39(+) | 0.23(-) | 0.43(-) |  |
| Fish and shellfish | 0.04(+) | 0.03(-) | 0.06(-) |  |
| Dietary fiber | 1x10^-5^(+) | 5x10^-3^(+) | 7x10^-3^(+) |  |
| Fruit and vegetables  Sucrose | 0.10(+)  0.80(-) | 0.92(-)  0.01(-) | 0.95(+)  0.07(-) |  |
| Alcohol habits | 1.00(+) | 0.08(-) | 0.046(-) |  |
| Smoking habits | 0.99(+) | 0.60(+) | 0.43(-) |  |
| Educational level | 0.38(-) | 0.58(-) | 0.66(-) |  |
| Leisure time physical activity  Diet changers in the past | 0.84(+)  6x10^-5^ (+)^4^ | 0.40(+)  0.06 (+) | 0.07(-)  0.09(+) |  |

^1^Test for trends using the General Linear Model with all variables treated as continuous variables, adjusted for

age and sex, *P* < 0.05. All continuous variables except age and the diet quality index were Ln-transformed.

^2^Direction of associations (+/-) with non-Ln transformed variables.

^3^Number of participants: BMI (n=24,770); smoking habits (n=24,789); educational level (n=24,743); leisure time

physical activity (n=24,647).

^4^Test for differences in number of diet changers and non-diet changers between tertiles of GRSs using Chi^2^ test

for categorical variables. Abbreviation: GRS, genetic risk score.
