# Supplementary material for: Genetic susceptibility to dyslipidemia and incidence of cardiovascular disease depending on a diet quality index in the Malmö Diet and Cancer cohort
Source: Genes Nutr. 2016 Jul 7;11:20. doi: 10.1186/s12263-016-0536-0 (PMC4968442; doi:10.1186/s12263-016-0536-0)
Supplement: Additional file 5: — Interaction (P value) between the genetic risk scores and the diet index components on incidence of total cardiovascular disease, coronary event, and ischemic stroke among 24,799 participants in the Malmö Diet and Cancer cohort. (DOCX 20 kb) [file 12263_2016_536_MOESM5_ESM.docx]

**Additional file 5:**

**Title:** Genetics susceptibility to dyslipidemia and incidence of cardiovascular disease depending on a diet quality index in the Malmö Diet and Cancer cohort.

**Journal name**: Genes and Nutrition

**Authors**: Sophie Hellstrand, Ulrika Ericson, Christina-Alexandra Schulz, Isabel Drake, Bo Gullberg, Bo Hedblad, Gunnar Engström, Marju Orho-Melander, Emily Sonestedt

**Affiliation**: Diabetes and Cardiovascular Disease – Genetic Epidemiology, Department of Clinical Sciences in Malmö, Lund University, Sweden

**Corresponding author**: sophie.hellstrand@med.lu.se

**Additional file 5**. Interaction (*P*-value) between the genetic risk scores and the diet index components on incidence of total cardiovascular disease, coronary

event and ischemic stroke among 24,799 participants in the Malmö Diet and Cancer cohort^1^

| Total CVD | Saturated fat | PUFA | Fish and shellfish | Dietary fiber | Fruit and vegetables | Sucrose |
| --- | --- | --- | --- | --- | --- | --- |
|  |  |  |  | *P*-interaction |  |  |
| GRS_LDL-C_  All  Men  Women | 0.35  0.46  0.56 | 0.22  0.82  0.13 | 0.95  0.31  0.26 | 0.50  0.47  0.12 | 0.38  0.11  0.84 | 0.76  0.80  0.94 |
| GRS_HDL-C_  All | 0.20 | 0.81 | 0.18 | 0.75 | 0.70 | 0.24 |
| Men  Women  GRS_TG_  All  Men  Women | 0.16  0.72  0.68  0.39  0.79 | 0.57  0.92  0.96  0.95  0.87 | 0.44  0.31  0.39  0.57  0.55 | 0.11  0.07  0.23  0.01  0.52 | 0.54  0.76  0.63  0.54  0.85 | 0.72  0.19  0.09  0.10  0.44 |
| Coronary event |  |  |  |  |  |  |
| GRS_LDL-C_  All  Men  Women | 0.81  0.38  0.19 | 0.62  0.76  0.73 | 0.50  0.25  0.66 | 0.07  0.92  0.01 | 0.30  0.67  0.40 | 0.83  0.97  0.84 |
| GRS_HDL-C_  All  Men  Women | 0.88  0.32  0.36 | 0.81  0.51  0.77 | 0.84  0.91  0.73 | 0.61  0.18  0.07 | 0.46  0.29  0.74 | 0.18  0.51  0.17 |
| GRS_TG_  All  Men  Women | 0.51  0.25  0.79 | 0.79  0.54  0.75 | 0.38  0.58  0.51 | 0.18  0.02  0.79 | 0.83  0.73  0.37 | 0.10  0.11  0.49 |
| Ischemic stroke |  |  |  |  |  |  |
| GRS_LDL-C_  All  Men  Women | 0.08  0.94  0.02 | 0.18  0.97  0.06 | 0.48  0.86  0.24 | 0.26  0.27  0.56 | 0.01  1x10^-3^  0.54 | 0.82  0.70  0.90 |
| GRS_HDL-C_  All  Men  Women | 0.06  0.31  0.12 | 0.90  0.96  0.90 | 0.07  0.15  0.27 | 0.88  0.34  0.49 | 0.77  0.70  0.91 | 0.85  0.79  0.62 |
| GRS_TG_  All  Men  Women | 0.92  0.92  0.98 | 0.65  0.51  0.99 | 0.72  0.79  0.84 | 0.80  0.29  0.58 | 0.63  0.13  0.52 | 0.52  0.58  0.66 |

^1^Cox proportional hazard regression was used to examine interactions (GRSs×diet index components as categorical variables) adjusted for age, sex, BMI, diet assessment method

version, season, total energy intake, alcohol habits, leisure time physical activity, educational level and smoking habits, *P* < 0.05. Abbreviation: GRS, genetic risk score.
