# Supplementary material for: Genetic susceptibility to dyslipidemia and incidence of cardiovascular disease depending on a diet quality index in the Malmö Diet and Cancer cohort
Source: Genes Nutr. 2016 Jul 7;11:20. doi: 10.1186/s12263-016-0536-0 (PMC4968442; doi:10.1186/s12263-016-0536-0)
Supplement: Additional file 6: — Hazard ratios for per 1 SD increase of the genetic risk scores in strata of diet quality index on incidence of total cardiovascular disease, coronary event, and ischemic stroke among 9383 men and 15,416 women in the Malmö Diet and Cancer cohort. (DOCX 19 kb) [file 12263_2016_536_MOESM6_ESM.docx]

**Additional file 6:**

**Title:** Genetics susceptibility to dyslipidemia and incidence of cardiovascular disease depending on a diet quality index in the Malmö Diet and Cancer cohort.

**Journal name**: Genes and Nutrition

**Authors**: Sophie Hellstrand, Ulrika Ericson, Christina-Alexandra Schulz, Isabel Drake, Bo Gullberg, Bo Hedblad, Gunnar Engström, Marju Orho-Melander, Emily Sonestedt

**Affiliation**: Diabetes and Cardiovascular Disease – Genetic Epidemiology, Department of Clinical Sciences in Malmö, Lund University, Sweden

**Corresponding author**: sophie.hellstrand@med.lu.se

**Additional file 6**. Hazard ratios for per 1 SD increase of the genetic risk scores in strata of diet quality index on incidence of total cardiovascular disease, coronary event and ischemic stroke among 9,383 men and 15,416 women in the Malmö Diet and Cancer cohort^1^

|  | Low  Men / Women | Diet quality index  Medium  Men / Women | High  Men / Women | *P*-interaction^2^ |
| --- | --- | --- | --- | --- |
|  | HR (95% CI) | HR (95% CI) | HR (95% CI) | Men/Women |
| Total CVD |  |  |  |  |
| GRS_LDL-C_ | 1.14 (1.02-1.28)/1.06 (0.93-1.22) | 1.07 (1.01-1.13)/1.12 (1.05-1.19) | 1.08 (0.93-1.26)/1.06 (0.91-1.22) | 0.43/0.61 |
| GRS_HDL-C_ | 1.09 (0.97-1.22)/1.08 (0.94-1.23) | 1.01 (0.96-1.07)/1.06 (0.99-1.13) | 1.06 (0.92-1.23)/1.13 (0.98-1.32) | 0.50/0.65 |
| GRS_TG_ | 1.05 (0.93-1.17)/1.00 (0.87-1.14) | 1.04 (0.98-1.10)/1.02 (0.96-1.09) | 1.00 (0.86-1.15)/1.11 (0.96-1.29) | 0.68/0.50 |
| Coronary event |  |  |  |  |
| GRS_LDL-C_ | 1.18 (1.03-1.35)/1.04 (0.86-1.25) | 1.05 (0.98-1.12)/1.14 (1.05-1.25) | 1.17 (0.98-1.41)/1.16 (0.95-1.41) | 0.87/0.19 |
| GRS_HDL-C_ | 1.02 (0.89-1.17)/1.04 (0.86-1.25) | 1.00 (0.94-1.08)/1.06 (0.97-1.16) | 1.03 (0.86-1.23)/1.24 (1.01-1.51) | 0.86/0.28 |
| GRS_TG_ | 1.07 (0.93-1.23)/1.06 (0.88-1.28) | 1.07 (1.00-1.15)/1.05 (0.96-1.15) | 1.03 (0.86-1.23)/1.18 (0.96-1.44) | 0.97/0.79 |
| Ischemic stroke |  |  |  |  |
| GRS_LDL-C_ | 1.07 (0.89-1.30)/1.09 (0.90-1.32) | 1.10 (1.00-1.20)/1.09 (0.99-1.20) | 0.92 (0.70-1.20)/0.94 (0.75-1.17) | 0.12/0.03 |
| GRS_HDL-C_ | 1.22 (1.00-1.48)/1.12 (0.92-1.35) | 1.02 (0.93-1.12)/1.05 (0.96-1.16) | 1.14 (0.89-1.47)/1.02 (0.82-1.27) | 0.16/0.62 |
| GRS_TG_ | 1.01 (0.83-1.22)/0.93 (0.77-1.13) | 0.99 (0.90-1.08)/0.99 (0.90-1.09) | 0.93 (0.72-1.20)/1.04 (0.83-1.30) | 0.48/0.55 |

^1^Cox proportional hazard regression was used to calculate HRs (95% CI) per 1 SD increase of the GRSs, *P* < 0.05, adjusted for age.

*^2^P*-interactions (GRSs×diet quality index as continuous variables) adjusted for age, BMI, diet assessment method version, season, total energy intake, alcohol habits,

leisure time physical activity, educational level and smoking habits. Abbreviations: CI, confidence interval; GRS, genetic risk score; HR, hazard ratio; SD, standard

deviation.
